# On optimal coupling of the ‘electronic photoreceptors’ into the degenerate retina

**DOI:** 10.1088/1741-2552/aba0d2

**Published:** 2020-07-24

**Authors:** Paul Werginz, Bing-Yi Wang, Zhijie Charles Chen, Daniel Palanker

**Affiliations:** 1 Institute for Analysis and Scientific Computing, Vienna University of Technology, Vienna, Austria; 2 Department of Physics, Stanford University, Stanford, CA, United States of America; 3 Hansen Experimental Physics Laboratory, Stanford University, Stanford, CA, United States of America; 4 Electrical Engineering, Stanford University, Stanford, CA, United States of America; 5 Ophthalmology, Stanford University, Stanford, CA, United States of America; 6 Author to whom any correspondence should be adressed.

**Keywords:** bipolar cell, ribbon synapse, neural stimulation, retinal prosthesis, electro-neural interface

## Abstract

**Objective.:**

To restore sight in atrophic age-related macular degeneration, the lost photoreceptors can be replaced with electronic implants, which replicate their two major functions: (1) converting light into an electric signal, and (2) transferring visual information to the secondary neurons in the retinal neural network—the bipolar cells (BC). We study the selectivity of BC activation by subretinal implants and dynamics of their response to pulsatile waveforms in order to optimize the electrical stimulation scheme such that retinal signal processing with ‘electronic photoreceptors’ remains as close to natural as possible.

**Approach.:**

A multicompartmental model of a BC was implemented to simulate responses of the voltage-gated calcium channels and subsequent synaptic vesicle release under continuous and pulsatile stimuli. We compared the predicted response under various frequencies, pulse durations, and alternating gratings to the corresponding experimental measurements. In addition, electric field was computed for various electrode configurations in a 3-d finite element model to assess the stimulation selectivity via spatial confinement of the field.

**Main results.:**

The modeled BC-mediated retinal responses were, in general, in good agreement with previously published experimental results. Kinetics of the calcium pumps and of the neurotransmitter release in ribbon synapses, which underpin the BC’s temporal filtering and rectifying functions, allow mimicking the natural BC response with high frequency pulsatile stimulation, thereby preserving features of the retinal signal processing, such as flicker fusion, adaptation to static stimuli and non-linear summation of subunits in receptive field. Selectivity of the BC stimulation while avoiding direct activation of the downstream neurons (amacrine and ganglion cells—RGCs) is improved with local return electrodes.

**Significance.:**

If the retinal neural network is preserved to a large extent in age-related macular degeneration, selective stimulation of BCs with proper spatial and temporal modulation of the extracellular electric field may retain many features of the natural retinal signal processing and hence allow highly functional restoration of sight.

## 1. Introduction

Phototransduction in photoreceptors (PRs) converts light into changes of the cell’s membrane potential, which then alters the release rate of the neurotransmitter glutamate into synapses of the secondary neurons—the bipolar cells (BCs). This affects the BC’s membrane potential, which defines the release rate of glutamate into synapses of the tertiary retinal neurons—the ganglion cells (RGCs). This input, in turn, modifies the RGC’s membrane potential, which governs its spiking rate—the visual signals subsequently propagate to the brain via the optic nerve. Synapses between PRs and BCs are regulated by inhibitory horizontal cells (HCs), while synapses between BCs and RGCs—by amacrine cells (ACs). Signal processing by the retinal neural network defines the way images on the retina are encoded in spiking patterns of the two dozen types of RGCs (Baden et al 2016).

In atrophic (dry) form of age-related macular degeneration (AMD), PRs gradually disappear in the central macula, but the inner retinal cells survive to a large extent (Kim et al 2002), albeit with some alterations (Jones et al 2016). To restore sight in the area of geographic atrophy, we replace the lost photoreceptors with photovoltaic pixels, which convert light into electric current to stimulate the secondary neurons in the retina (Lorach et al 2015a, Palanker et al 2020). These ‘electronic photoreceptors’ replace the two main functions of the natural photoreceptors: (1) the light-to-current conversion, corresponding to the function of the photoreceptor outer segment, and (2) conveying the visual information to secondary neurons by the generated extracellular electric field—an electronic substitute of a synapse. In this paper, we discuss the possibilities and limitations of proper coupling of these ‘electronic photoreceptors’ into the retinal neural network in order to preserve the remaining signal processing to the maximum extent possible.

Selective stimulation of bipolar cells without direct activation of the downstream neurons, the amacrine and ganglion cells, can be achieved by utilizing the differences in cellular shape and location as well as ion channels expressed in various types of retinal neurons. For this purpose, we model the electric field with various configurations of subretinal electrodes as well as the response of BCs and RGCs to pulsed electrical stimulation and compare it to experimental measurements.

Since conductivity in metals and semiconductors is electronic, while in liquids it is ionic, transfer of the electric current from an electrode to an electrolyte can be mediated by either the double layer capacitance or by electrochemical reactions. To avoid irreversible electrochemistry, which may lead to electrode erosion and/or tissue damage, the applied current should be pulsed and charge balanced. On the other hand, to provide steady visual percepts under pulsed illumination, the pulse repetition rate should exceed the frequency of flicker fusion. We discuss the cellular machinery responsible for the signal processing in BCs—the voltage-sensitive ion channels, calcium pumps and ribbon synapses—and demonstrate that many of its functions are largely preserved under pulsed electrical stimulation. In particular, we show how the temporal filtering and non-linearity of the ribbon synapses between BCs and RGCs retain the functions of flicker fusion and adaptation under electrical stimulation.

We review the consequences of selective activation of BCs and experimental evidence of preservation of multiple features of the natural signal processing with a subretinal prosthesis, including antagonistic center-surround organization of receptive fields and non-linear summation of its subunits, as well as limitations on selectivity in the activation of the ON and OFF pathways in the retina.

This paper consists of three parts: (1) First, we describe the computational models for electrical activation of BCs and RGCs, and assess the selectivity of BC stimulation by a point source of electric current; (2) Then we show that better spatial confinement of the electric field can further increase selectivity of the BC activation; (3) Finally, we compare the temporal response of BCs under pulsatile stimulation to that with a continuous stimulus and discuss the cellular mechanisms and consequences of flicker fusion.

## 2. Methods

### 2.1. Modeling the extracellular potential

**Figure 1.**
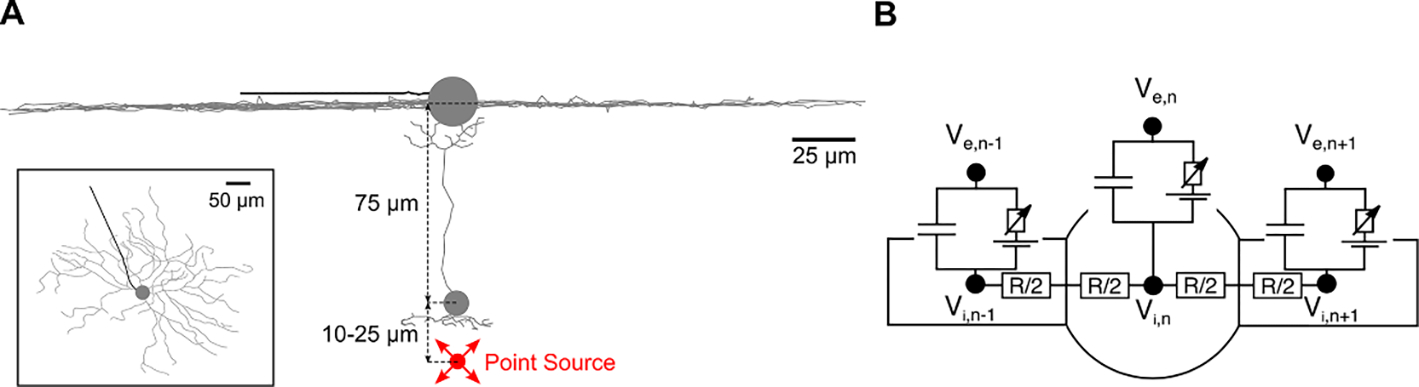
Subretinal stimulation with a point source. (**A**)Side view of the degenerate retina with one RGC and one BC. A point source electrode (red) located in the outer plexiform layer. The inset shows the top view of the model RGC. (**B**) Schematic of the electric circuit of multicompartmental models. Membrane potential was defined as $V=V_{i}-V_{e}$.

To compare the computed stimulation thresholds with experimental measurements performed using a micro-pipette electrode for stimulation (Boinagrov et al 2014), we modeled the extracellular potential for a point source placed in the outer plexiform layer (OPL), as shown in figure 1(A). The extracellular potential distribution $\left(V_{e}\right)$ generated by an ideal point source electrode in homogenous medium was modeled as $V_{e}=\frac{I_{\text{stim}}*\rho_{\text{ext}}}{4^{⋆}\pi^{⋆}r}$, with $I_{\text{stim}}$ being the applied current, $\rho_{\text{ext}}=1000\;\Omega \;cm$ being the resistivity of the surrounding medium (the retina (Werginz and Rattay 2016)), and r being the Euclidean distance from the electrode to each cellular compartment (see below).

To simulate responses of BCs and RGCs to extracellular electrical stimulation, we developed multicompartmental models of BCs and RGCs with realistic anatomy. Current flow along the intracellular space as well as across the cell membrane is simulated by a network of compartments with given electrical properties (Rall 1964, Rattay 1999). Figure 1(B) shows the electric circuit of a multicompartmental model, the change of membrane potential $\left(dV_{n}\right)$ at each time step was described by

$$\frac{dV_{n}}{dt}=\left[-I_{\text{ion},n}+\frac{V_{n-1}-V_{n}}{\frac{R_{n-1}}{2}+\frac{R_{n}}{2}}+\frac{V_{n+1}-V_{n}}{\frac{R_{n+1}}{2}+\frac{R_{n}}{2}}+\cdots \right.\;\;\left.+\frac{V_{e,n-1}-V_{e,n}}{\frac{R_{n-1}}{2}+\frac{R_{n}}{2}}+\frac{V_{e,n+1}-V_{e,n}}{\frac{R_{n+1}}{2}+\frac{R_{n}}{2}}+\cdots \right]*\frac{1}{C_{n}}$$

with $I_{\text{ion}}$ being the ionic current across the membrane which was computed by the dynamics of Fohlmeister (Fohlmeister et al 2010) for RGCs and a simplified membrane model for BCs (see below). Currents between neighboring compartments were dependent on each compartment’s intracellular resistance (R) and the rate of change of membrane potential was dependent on the membrane capacitance (C). The influence of extracellular electrical stimulation $\left(V_{e}\right)$ was coupled into the model via the activating function (Rattay 1999). The dots in the equation indicate the possibility of more than two neighboring compartments.

### 2.2. Ganglion cell model

RGC membrane dynamics were modeled as previously presented by Fohlmeister et al (Fohlmeister et al 2010) as this model was shown to capture the main characteristics of the RGC responses observed in intracellular stimulation experiments. The ion channel densities are listed in table 1 for the five different sections along the neuron: dendrites, soma, axon hillock (soma-AIS), axon initial segment (AIS), and the axon. Intracellular resistivity was set to 143.2 Ω cm and specific membrane capacitance to 1 *μ*F cm^−2^. The cell morphology of a traced mouse ON-α sustained RGC (Raghuram et al 2019), shown in figure 1(A), consisted of 2182 compartments, with the compartment length ranging from 3 to 5 *μ*m.

### 2.3. Bipolar cell model

The simplified bipolar cell model has been adjusted from a previous study (Werginz and Rattay 2016). In brief, the 2-d morphology of a rat ON bipolar cell has been extracted from the literature (Euler and Wässle 1995, Encke et al 2013) and has been converted into a realistic 3-d multicompartmental model (figure 1(A)). We used an anatomical reconstruction of a rat BC (in comparison to the mouse RGC) as we are not aware of detailed reconstructions of mouse BCs. However, results presented in this study are not dependent on detailed morphological features which were also shown to be similar between mouse and rat retina (Ghosh et al 2004).

A linear leak current was assumed to be distributed over the whole neural membrane, and a calcium L-type channel was localized to synaptic terminals only. Ionic current densities at the n^th^ compartment were described by the following set of equations:

$$i_{\text{ion},n}=i_{CaL,n}+i_{L,n},\;\text{with}\;\;\;i_{CaL,n}=g_{Ca,n}*m_{n}^{2}*h_{n}*\left(V_{n}-E_{Ca,n}\right)\;\;i_{L,n}=g_{L,n}*\left(V_{n}-E_{L}\right)$$

Ion channel densities of the calcium $\left(g_{Ca,n}\right)$ and leak channel ${(g}_{L,n})$ for the four different sections (dendrites, soma, axon, axon terminals) are listed in table 2. The leak reversal potential $\left(E_{L}\right)$ as well as the resting membrane potential were assumed to be −60 mV (Walston et al 2018), while the reversal potential for calcium $\left(E_{Ca,n}\right)$ was computed as a Nernst potential based on the intracellular calcium concentration [Ca]_i_:

$$E_{Ca,n}=10^{3}*\frac{R*T}{2*F}*ln\frac{[Ca{]}_{e}}{[Ca{]}_{i,n}}$$

with the gas constant $R=8.31\;J/K^{*}\;mol$, temperature T=296.15K, Faraday’s constant $F=9.6485^{*}10^{4\;}C{\;mol}^{-1}$, the intracellular calcium concentration $[Ca{]}_{i,n}$ (see below for calculation) and the extracellular calcium concentration $[Ca{]}_{e}=1800\;\mu M$.

Intracellular resistivity was set to 130Ωcm, specific membrane capacitance to 1 *μ*F cm^−2^. The L-type calcium channel model presented previously (Werginz et al 2015) was adjusted to account for slightly slower activation kinetics. Gating variables m and h were defined by the following equations:

$$\frac{dm}{dt}\;=\alpha_{m}*(1-m)-\beta_{m}*m,\;\text{with}\;\;\alpha_{m}\;=\frac{0.21*(V+5)}{1-exp\left(-\frac{V+5}{10.5}\right)}\;\text{and}\;\beta_{m}=0.02*exp\left(\frac{12-V}{12}\right)$$

$$\frac{dh}{dt}=\frac{h_{\infty }-h}{\tau_{h}},\;\text{with}\;\;h_{\infty }=\frac{1}{1+exp\left(\frac{V+55}{66.4}\right)}\;\text{and}\;\tau_{h}=292\;ms$$

Dependence of the intracellular calcium concentration $\left([Ca{]}_{i,n}\right)$ on influx of calcium ions through the calcium channel and on passive extrusion was defined as following:

$$\frac{d[Ca{]}_{i,n}}{dt}=-\frac{i_{Ca,n}}{2*F*d}-\frac{[Ca{]}_{i,n-}[Ca{]}_{res}}{\tau }$$

with the depth at which $[Ca{]}_{i,n}$ was determined d=50nm, the residual calcium level $[Ca{]}_{res}=0.1\;\mu M$, and the time constant of the extrusion process τ=50ms.

### 2.4. Synaptic release model

Release of neurotransmitter-filled vesicles from the ribbon synapses was modeled similarly to a previously published model (Sikora et al 2005), with the release rate dependent on the intracellular calcium concentration in BC terminals as well as on the current vesicle filling state of each synapse. In brief, vesicles can be released from two separate vesicle pools: (1) a fast pool, which represents vesicles already primed for release, and (2) a slow pool with vesicles that are not readily releasable. The mathematical framework to compute vesicle release has been adapted from Sikora et al (Sikora et al 2005). The model converts intracellular calcium concentration in BC terminals to an instantaneous release rate. This vesicle release rate is further applied independently and stochastically to each release site of the synaptic ribbon. We slightly modified the original model by using the intracellular calcium concentration at only one depth (50 nm) for both vesicle pools. We compared the release model against data from a previous study showing strong transient release emptying the fast pool within 20 ms, followed by sustained steady-state release of approximately 0.5 vesicles/ms/terminal when the BC was depolarized from −60 to −10 mV (Singer and Diamond 2006). A manual fitting procedure was performed to match experimental data. The best fit was obtained by slowing the calcium-dependent rate constant for the fast pool down by a factor of 20 and for the slow pool by a factor of 800 (in comparison to (Sikora et al 2005)). Each synaptic terminal (n = 8) was equipped with 10 ribbon synapses, resulting in a total number of 80 synapses per BC. 6 release sites with 5 rows of vesicles were defined at each synapse, resulting in a total number of 30 releasable vesicles per synapse. Vesicles could be refilled to the synapse at a time constant of 1 s, except otherwise noted, similar to experimental results (Singer and Diamond 2006).

All models were solved in Matlab (Mathworks) using a custom written implicit (backward) Euler solver. Time step was set to 0.025 ms. Since we compared modeled results primarily to data obtained in ex-vivo experiments at room temperature, model temperature was set to 23 °C in all simulations.

### 2.5. Modeling the electric field

In addition, we calculated the electric field in an electrolyte using a 3-d finite element model of complete arrays of approximately 1000 pixels in COMSOL Multiphysics 5.4. The electrostatics module was implemented to solve Poisson’s equation for electrical conduction, assuming a steady-state electric current of 0.2 *μ*A, close to the stimulation threshold with 40 *μ*m pixels (Flores et al 2019). To illustrate the differences in field confinement among various pixel designs, we computed the electric field potential distribution for four distinct geometric configurations of the active (anodic) and return (cathodic) electrodes: flat monopolar (common return at the edge of the chip) and 3 bipolar (local returns surrounding each pixel)—flat, pillar and honeycomb (figure 3). All modeled arrays are 1.5 mm in diameter, 30 *μ*m thick, and consist of 40 *μ*m hexagonal pixels with an active electrode of 16 *μ*m in diameter. In bipolar pixels (flat and 3-D) the return electrode was 4 *μ*m in width, surrounding each active electrode in a hexagonal array. Pillar electrodes were 10 *μ*m in height, and honeycombs were 25 *μ*m in height, as described in our previous publications (Flores et al 2019, Ho et al 2019).

The thickness of the degenerate retina was assumed to be 100 *μ*m, and the resistivity of the medium in that layer was assumed to be equal to that of the retina: 1000 Ω cm (Werginz and Rattay 2016), while above it—that of the vitreous: 90 Ω cm (Oksala and Lehtinen 1959). To model the steady state behavior, constant current density is injected from active electrodes and collected on return electrodes (Chen et al 2020). The array of pixels is enclosed in a 1 cm^3^ cubic volume, where the peripheral sides of the cube are defined as the ground. The initial values are set such that the total current injected from active electrodes is equal to the current collected at designated return electrodes. The bounding box is much larger than the implant modeled, so the gradient of electric potential at the boundary is negligible. Source of and sink of electric current are assumed to be confined to the electrode array, as in photovoltaic implants, and therefore the electric potential on active electrodes is above, and on return electrodes is below the ground level during the pulse.

## 3. Results

### 3.1. Factors shaping the chronaxies in BCs

To compare our modeling results to measurements performed with a micro-pipette electrode (Boinagrov et al 2014), we simulated BC and RGC responses to extracellular stimulation defined by a point source electrode located in the outer plexiform layer (OPL, electrode 15 *μ*m from BC soma) and applied monophasic, anodic stimulation (figure 1(A)). Threshold for RGC activation during the anodic phase of the pulse was defined as the minimum stimulus amplitude that elicited an action potential that propagated as far as the distal axon (~950 *μ*m from soma) within 3 ms after the pulse offset. Indirect RGC responses are mediated by synaptic activity in BC terminals, which is driven by elevated intracellular calcium concentration ([Ca]_i_) and subsequent vesicle release. The BC threshold was defined as a release of 3 vesicles from the synapse. Since the cathodic phase of the charge-balanced pulse in photovoltaic stimulation lasts several times longer than the anodic phase, with accordingly lower amplitude (Boinagrov et al 2016), and stimulation threshold during the cathodic phase is about 4 times higher than in the anodic phase (Boinagrov et al 2014), we did not consider the cathodic phase in retinal stimulation here.

**Figure 2.**
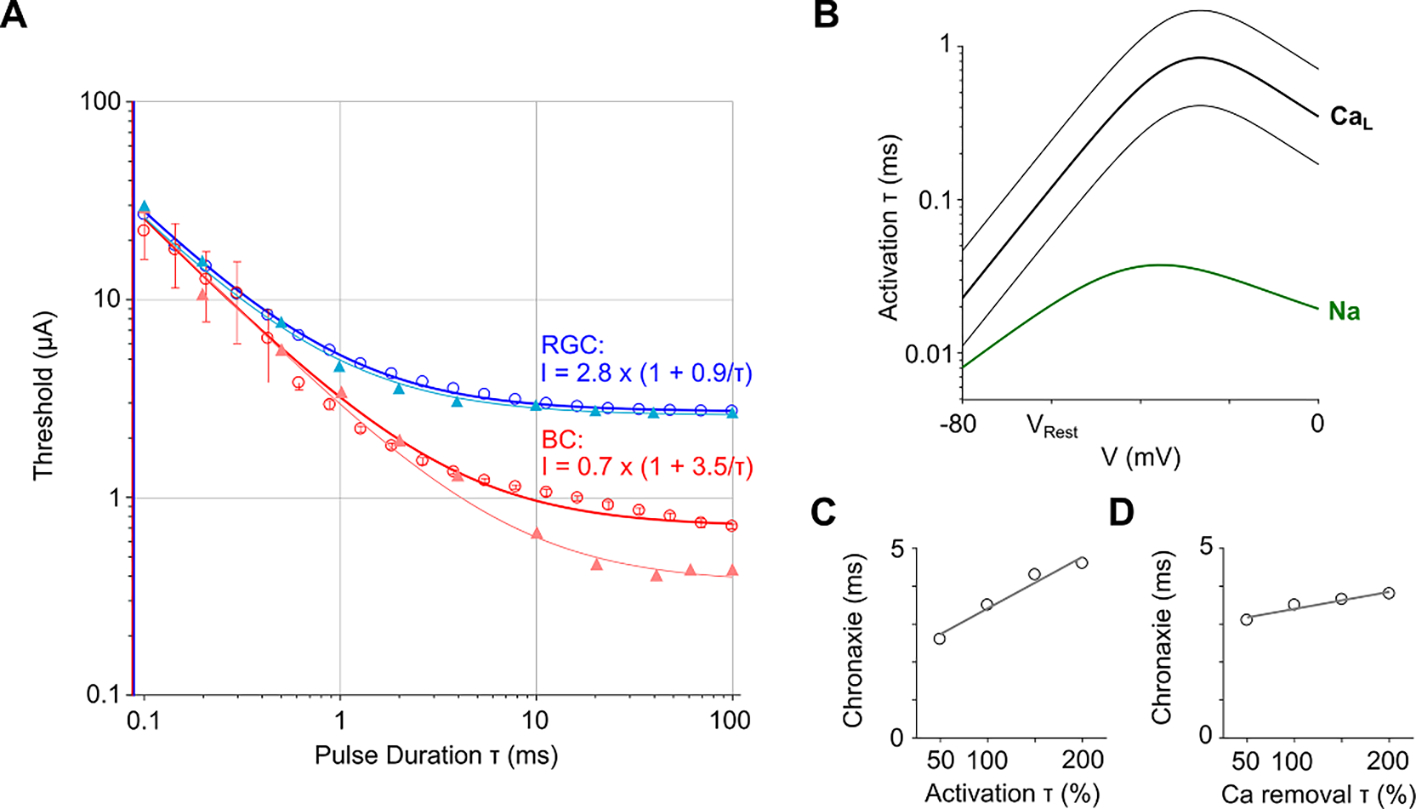
Strength-Duration (S-D) curves for BC and RGC activation. (**A**) Calculated thresholds for stimulation of BCs (red circles) and RGCs (blue circles), fitted with Weiss equation, solid lines. BC activation was based on the average vesicle release from 20 repetitions of each simulation. Experimental data from (Boinagrov et al 2014) is shown by triangular markers. Error bars indicate one standard deviation. (**B**) Activation time constants of the voltage-gated L-type calcium (thick black line) and sodium (green) channels. Thin lines indicate halved and doubled activation time constant of the calcium channel. (**C**) BC chronaxie as a function of the calcium channel activation time constant. (**D**) BC chronaxie as a function of the calcium removal time constant. Gray solid lines in (C) and (D) indicate linear regressions.

The computed Strength-Duration (S-D) curves for RGCs (blue) and BCs (red) in the range of pulse durations from 0.1 to 100 ms are shown in figure 2(A). The simulated thresholds for RGCs matched the experimental values very closely (blue). Since the vesicles release is a stochastic process, the BC model required multiple runs for each pulse duration to compute an average number of released vesicles. Figure 2(A) (red) shows the S-D curve for 20 repetitions of the BC activation. As in earlier experimental measurements (Boinagrov et al 2014), BC thresholds decreased monotonically with increasing pulse duration up to a rheobase, and could be well-fit by Weiss equation (Weiss 1901). Modeling confirmed much longer chronaxie for BCs (>3 ms) than RGCs (<1 ms), and therefore longer pulses can preferentially activate BCs over RGCs.

Simulated BC thresholds matched the experimental data well for short pulse durations (0.1–2 ms), but did not decrease as much at longer pulses (figure 2(A)). Consequently, the rheobase in simulations was not as low as in experimental data (0.7 vs. 0.4 *μ*A). BC thresholds (including the rheobase) could be lowered by (1) increasing the extracellular resistivity of the surrounding medium, (2) increasing the calcium channel density on BC terminals, or (3) lowering the threshold criterion. However, these modifications affect the whole curve in the same way, i.e. a better fit at long pulse durations results in underestimation for short pulses. The S-D curve with BC activation defined by elevated [Ca]_i_ did not fit the Weiss equation as well as that based on the vesicles release, and was even lower at pulse durations below 1 ms (see figure S1) (available online at stacks.iop.org/JNE/17/045008/mmedia).

In order to initiate an action potential in RGCs, voltage-gated sodium channels should open during depolarization and remain open long enough for the membrane potential to rise to the threshold value. This opening occurs in tens of microseconds (figure 2(B), green), which results in the observed sharp onset of spikes. On the other hand, RGC responses mediated by the retinal network require synaptic input from the stimulated BCs. This activity is driven by the voltage-gated calcium channels located in the pre-synaptic terminals of BCs. Calcium channels are about 1–2 orders of magnitude slower than the sodium channels—their opening time constant is around 1 ms (figure 2(B), black).

To assess the effect of various model parameters on chronaxie for BC activation, we first modified the activation time constant of the calcium channel (figure 2(B)). Chronaxie extracted from the computed S-D curves increased linearly with the activation time constant (figure 2(C)). Similarly, the calcium removal time constant, which determines the passive extrusion of [Ca]_i_ from the intracellular space also affected chronaxie for BC activation in a linear fashion (figure 2(D)), i.e. slower removal of [Ca]_i_ increased the chronaxie. However, the effect of the Ca extrusion rate on chronaxie was much smaller than that of the Ca channel activation time. On the other hand, neither the threshold criteria for BC activation nor the calcium channel density on BC terminals affected the chronaxie since the resulting S-D curves just shifted along the threshold (vertical) axis without changing their curvature (not shown).

### 3.2. Effect of the local return electrode on shaping the electric field

Since the cell cytoplasm is much more conductive than the cell membrane, upon application of an electric field (current) in the surrounding medium, the cell quickly polarizes by redistributing electric charges along its membrane. Since cations are attracted to the anode (area of higher potential) and anions—to the cathode (lower potential), the cell polarizes such that the negative membrane potential on its anodic side increases (membrane is hyperpolarized), while on the cathodic side, it decreases (membrane is depolarized). With the anode located in the OPL, the soma and dendritic parts become hyperpolarized, while the axonal portion of the BC becomes depolarized. Opening of the voltage-sensitive Ca ion channels in axonal terminals of the BC in response to depolarization consequently triggers the release of synaptic vesicles (as reviewed in (Wan and Heidelberger 2011)).

To assess how the shape of the electric field affects the BC stimulation threshold, we compared membrane polarization obtained with a point source to that observed in a uniform electric field that had the same total voltage drop between the top and bottom (z-dimension) of the BC (figure S2(A)). This comparison demonstrated that the uniform field created slightly (by about 25%, range 18%–31%) larger depolarization of the axonal terminals, both of which scale linearly with electric field (figures S2(B)–(C)). Therefore, BC membrane polarization can be estimated for various field shapes by just the voltage step between its top and bottom boundary.

**Figure 3.**
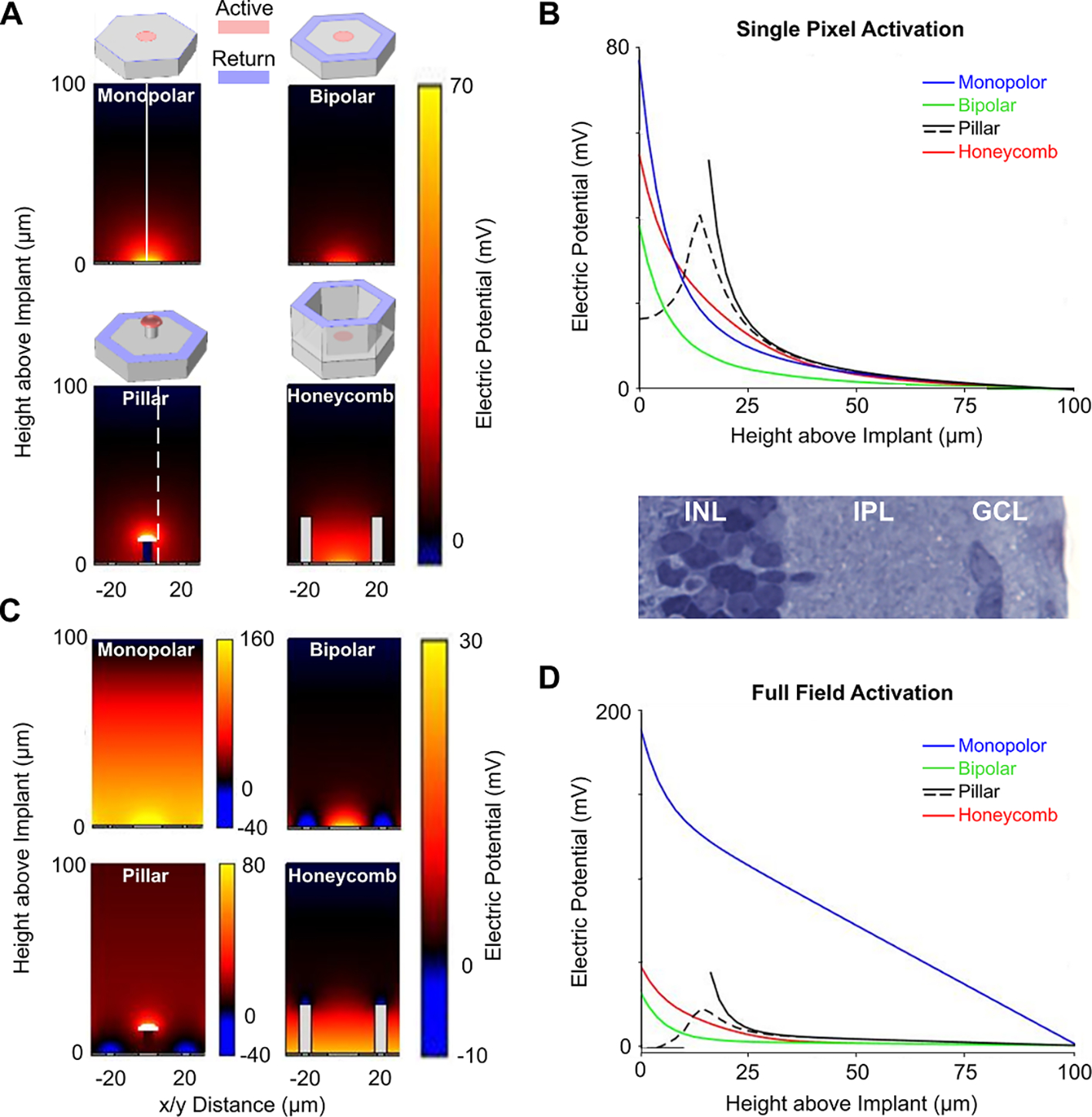
Electric potential in the retina with various electrode configurations. (**A**) Electric potential in the medium of 1000 Ω cm resistivity, generated by 0.2 *μ*A of current flowing from the active electrode in steady state, when a single pixel is activated in the array. The active electrode is shown in red, and the return—in blue. (**B**) Electric potential as a function of z-distance on axis (solid vertical line in (A)) or slightly off axis (dashed vertical line in (A)) of the pixel. (**C**) Electric potential in the medium when all pixels in the array are activated by the same current (0.2 *μ*A) simultaneously. (**D**) Corresponding electric potential as a function of z-distance on axis of the pixel. Rat retinal histology is shown to scale with the plots in (B) and (D); INL: inner nuclear layer, IPL: inner plexiform layer, GCL: ganglion cell layer. All potentials are calculated relative to the medium above the retina (Height above Implant = 100 *μ*m).

To provide selective stimulation of BCs while avoiding activation of the downstream neurons—ACs and RGCs, the electric potential should rapidly drop across the INL. As shown in figures 3(A)–(B), activation of a single monopolar pixel (with a distant return electrode) generates an electric potential which declines across the retina with a rate similar to that in a bipolar pixel (where a local return electrode surrounds an active electrode). However, when many pixels are activated simultaneously, electric fields from monopolar electrodes add up, resulting in a much slower decline in the potential across the retina. Such a deep penetrating field provides much lower selectivity in activation of BCs vs. ACs, which are located in the top (inner) strata of INL (Balasubramanian and Gan, 2014), than with bipolar pixels (figures 3(C)–(D)). In addition, since the electric potential in front of the monopolar array increases with the number of activated pixels, perceptual brightness of the image will depend on its sparsity. Local return electrodes in bipolar pixels isolate their electric fields from the neighbors, and hence the perceptual brightness should not depend on the image sparsity.

However, as we demonstrated earlier (Flores et al 2019), retinal stimulation thresholds with flat bipolar pixels below 40 *μ*m in width exceed the charge density limit even for SIROF electrodes. To overcome this limitation, two 3-dimensional configurations have been proposed: pillar electrodes (Ho et al 2019) and honeycomb arrays (Flores et al 2019). Both approaches utilize the phenomenon of cellular migration in the subretinal space, when the cell somas in the INL fill the space provided in the subretinal implant over a few weeks post implantation. As a result, pillar (active) electrodes penetrate into the middle of INL, which helps reduce the stimulation threshold (Ho et al 2019). However, as the maximum electric potential is shifted closer to the top of INL, the discrimination between BCs and ACs is likely weakened. With honeycomb arrays, the penetration of electric field into the INL is also increased via elevating the return electrode, but the maximum potential is located at the bottom, which likely provides better selectivity. Other advantages of the honeycomb arrays in size scalability and in contrast are described in the previous publication (Flores et al 2019).

### 3.3. BC response to high frequency stimulation—the low-pass filtering

**Figure 4.**
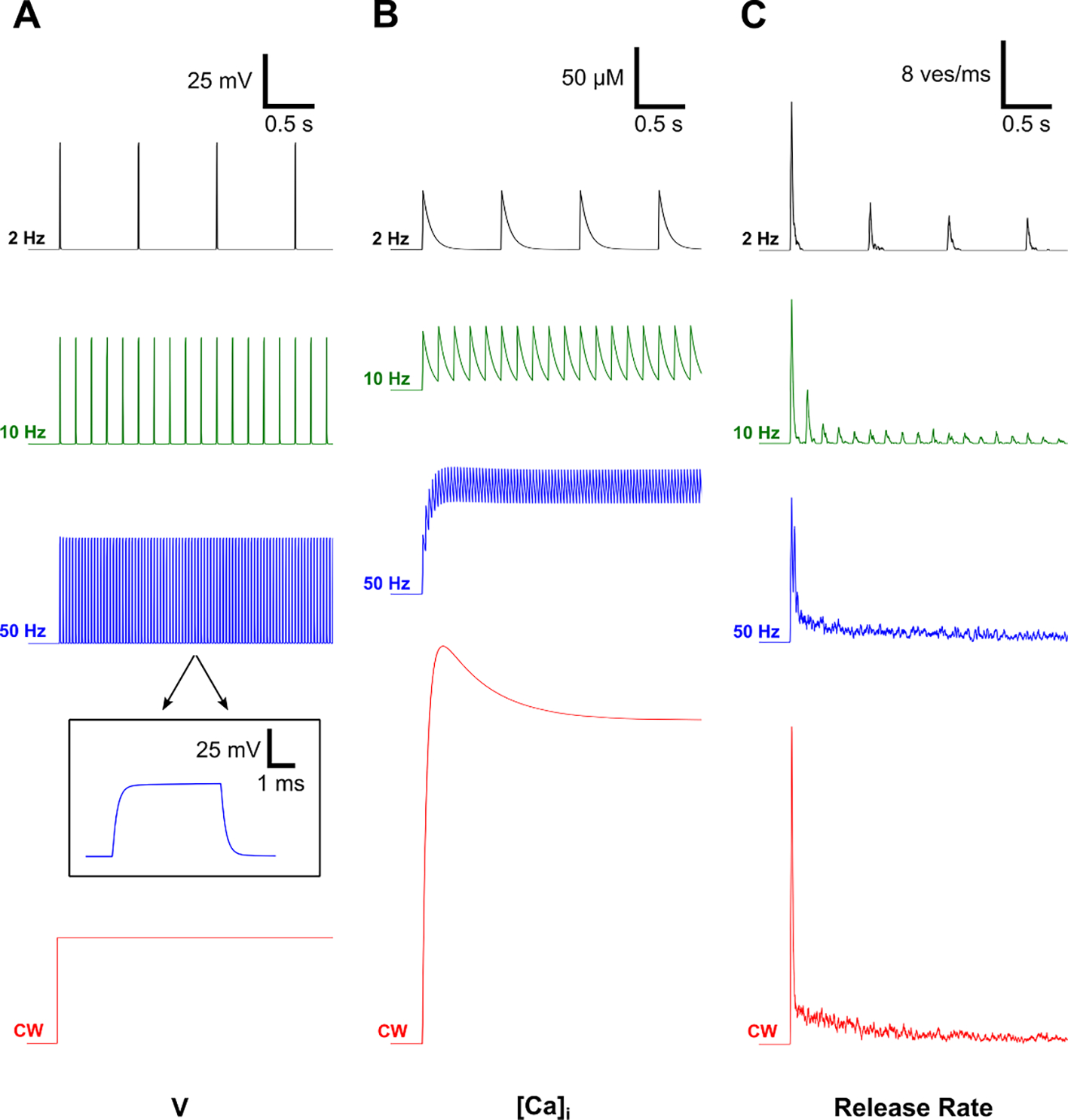
BC activation and vesicle release during pulse train stimulation. (**A**) Membrane potential (V) in BC terminals for pulsatile stimulation of 4 ms in duration and frequencies ranging from 2 to 50 Hz, as well as continuous wave (CW) stimulation. The inset shows the membrane potential on a finer time scale. (**B**) Intracellular calcium concentration [Ca]_i_. (**C**) Average release rate in 8 BC terminals at various frequencies.

**Figure 5.**
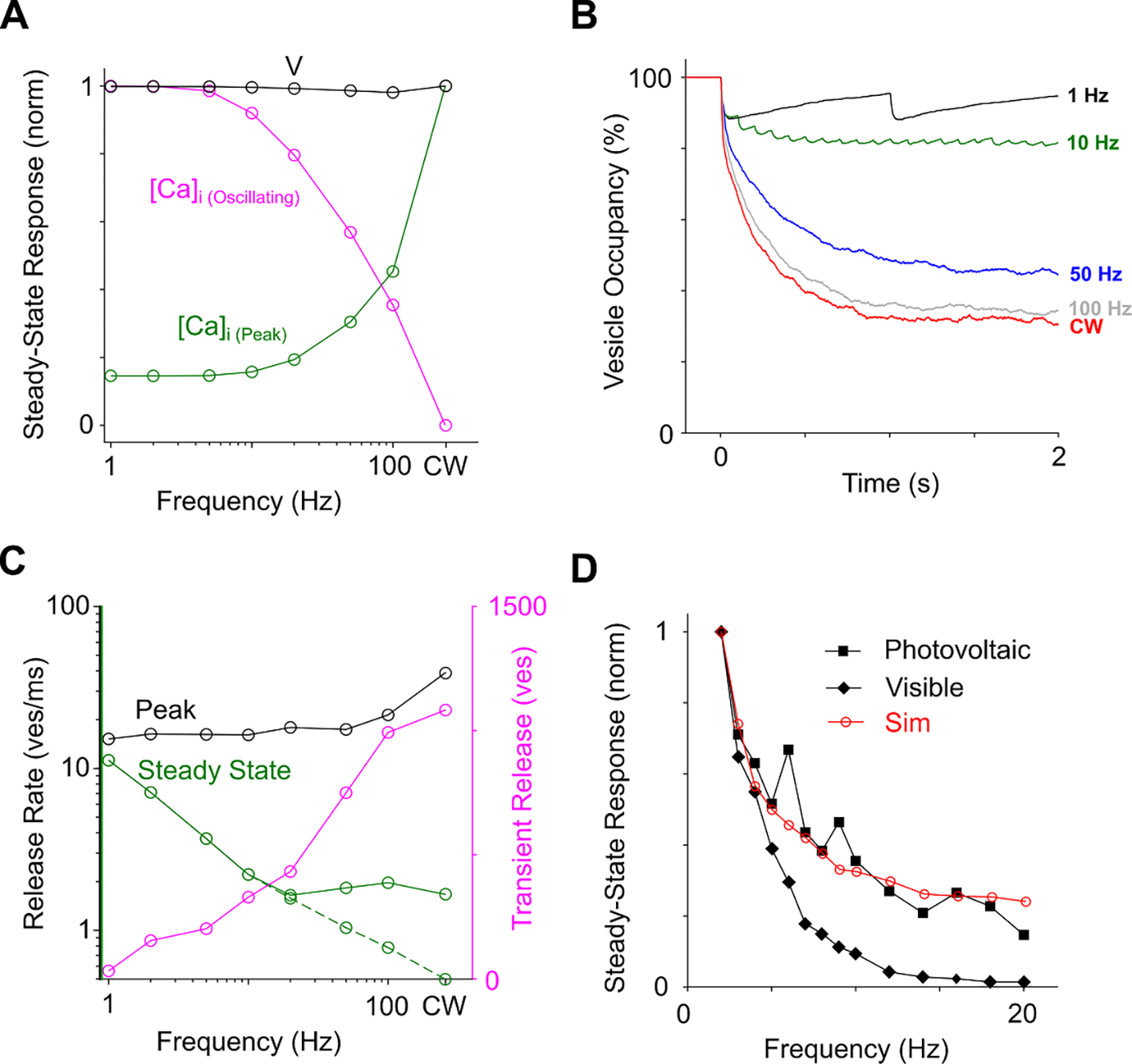
Ca concentration and vesicles release characteristics during pulse train stimulation. (**A**) Peak V (black), peak [Ca]_i_ (dark green) and the amplitude of oscillation of [Ca]_i_ (magenta) for different stimulus frequencies as well as continuous wave stimulation (CW). (**B**) Vesicle occupancy over time for 1, 10, 50 and 100 Hz as well as CW. Data is averaged over 80 ribbon synapses. (**C**) Peak release rate (black), steady-state release rate (green) and number of released vesicles within one second (magenta) for pulsatile (1–100 Hz) and CW stimulation. Release rate for each pulse in steady state was measured as a mean value (solid green line) and as an oscillatory value (dashed green line). (**D**) Vesicles release rate (red, same data as figure 3(C) green dash line) compared to the RGC firing rate measured ex-vivo during photovoltaic stimulation of degenerate retina (black squares) and visible light stimulation of normal retina (black diamonds) from (Lorach et al 2015a).

Membrane depolarization in BC synaptic terminals in response to repetitive pulsed stimuli (monophasic, anodic pulses at 4 ms and 4 *μ*A from a point source in the OPL, see Methods) is shown in figure 4(A). Responses to each pulse at frequencies ranging from 2 to 50 Hz did not change since the cell can be readily de- and re-polarized by the applied electric field with a time constant of about 0.2 ms (figure 4(A), inset). Subsequently, membrane depolarization led to opening of the voltage-sensitive calcium channels in the synaptic terminals, which resulted in an inward calcium current (not shown) leading to increased calcium concentration [Ca]_i_ (figure 4(B)). Since upon opening of the Ca channels the ions can only flow into the cell (due to the voltage and concentration difference), the cell acts as a rectifier—converting the AC stimulus into the pulsatile inward current. After the pulse, calcium channels close and ions are removed from the intracellular space by ion pumps with a time constant of 50 ms. At low frequencies (<5 Hz), the inter-pulse interval was long enough to let [Ca]_i_ return to its resting state before the next stimulus. Higher pulse rates (10–100 Hz), on the other hand, led to an elevated level of [Ca]_i_ even between the pulses. Therefore, the amplitude of the oscillation in [Ca]_i_ concentration at steady state decreased with increasing frequency, while the constantly elevated level increased (figures 4(B) and 5(A)). Such a low-pass filtering in BCs may be partially responsible for the flicker fusion phenomenon—diminished perception of pulsation with increasing frequency of the flash stimuli. The other part of such filtering occurs in synaptic transmission, as described in the next section.

### 3.4. Synaptic kinetics results in high-pass and low-pass filtering of the BC output

Increased [Ca]_i_ drives the release of the neurotransmitter-filled vesicles from ribbon synapses in BC terminals in a biphasic manner, with a fast transient release phase followed by a sustained release at lower rate (Singer and Diamond 2003, Oesch and Diamond 2011). Since availability of the readily releasable vesicles decreases after the first transient release, the following pulses have attenuated amplitude, and this attenuation increases with the pulse frequency (figure 4(C)). To better understand the kinetics of the vesicle release, we plotted the average vesicle occupancy of 80 synapses over the initial 2 s of the stimulus (figure 5(B)). The average filling state decreased from about 80% at 10 Hz to lower levels as the stimulation frequency increased to 50 and 100 Hz—closer to the level of continuous stimulation (red).

Figure 5(C) demonstrates that the peak release rate, i.e. the maximum release rate during the whole stimulus, starts increasing with frequency above 50 Hz due to summation of a few pulses within the rapid release phase of the ribbon synapse. We also characterized responses based on the oscillatory release rate which was defined as the difference between the maximum release rate and the minimum release rate during each pulse. The oscillatory release rate in steady-state monotonically decreases with frequency, as shown in figure 5(C) (dash green line). On the other hand, the average release rate decreases with frequency up to about 20 Hz, and then stabilizes on a constant level, as shown by a solid green line in figure 5(C). Integrating the difference between the transient and steady-state release rate over time provides the total number of vesicles released in response to a step function illustrated in figure 4(C). As shown in figure 5(C), this value increases with frequency as well.

Assuming that the RGC firing rate is proportional to synaptic input in pulsed stimulation, we can compare predictions of our model to experimental data. Figure 5(D) depicts the population-averaged RGCs firing rate as a function of the stimulation frequency in two experiments: degenerate rat retina (Royal College of Surgeons (RCS) rats) stimulated by a subretinal prosthesis and normal retina (Long Evans rats) stimulated by visible light (Lorach et al 2015a), together with the vesicle release rate in steady state from the current model. Results of our model show that the declining response with increasing frequency is similar to the decrease observed in the degenerate retina, while in normal retina, the response declines much faster. The matching rate of this decrease between our model and degenerate retina suggests that bipolar cells are the main temporal filter in this case (Ca accumulation and vesicle release from BC terminals). The faster decline in normal retina indicates that photoreceptors (phototransduction and ribbon synapse) represent another temporal low-pass filter, on top of the filter by bipolar cells, which matches the previous direct measurements of the photoreceptors response (Pandarinath et al 2010).

**Figure 6.**
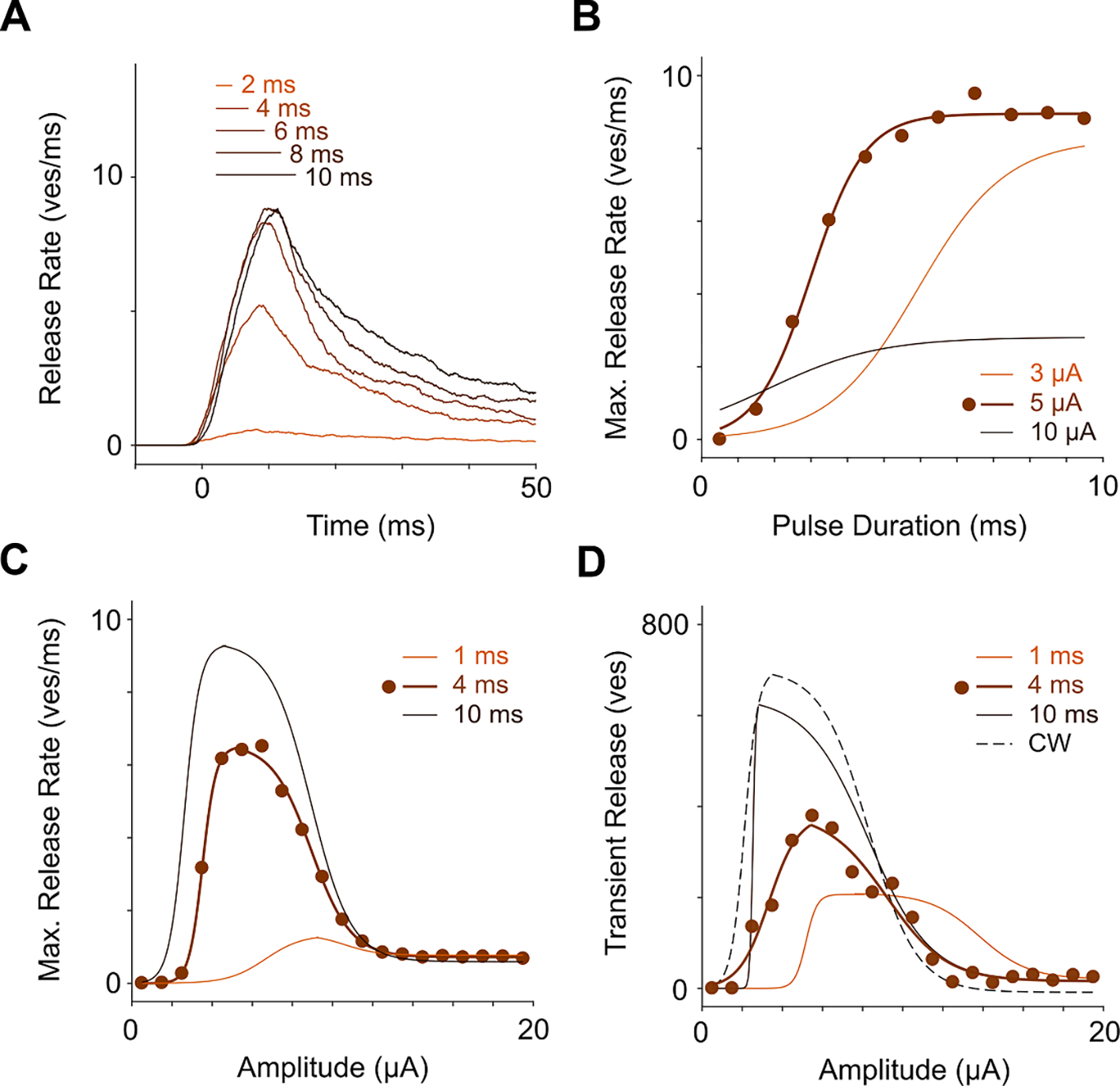
Vesicle release rate as a function of stimulus amplitude and duration. (**A**) Vesicle release during a 2 Hz pulse train (4 *μ*A) for different pulse durations. (**B**) Maximum vesicle release rate for different pulse durations and amplitudes. Solid lines indicate sigmoid fits for different amplitudes. Individual data points are only shown for 5 *μ*A stimulation. (**C**) Maximum vesicle release rate for different amplitudes and pulse durations. Individual data points are only shown for 4 ms stimulation. (**D**) Transient release integrated over one grating cycle (500 ms) as a function of amplitude for a grating reversal at 50 Hz carrier frequency with different pulse durations, as well as with continuous wave stimulation (CW).

Another important aspect of the BC response is the effect of pulse duration on vesicle release. Figure 6(A) shows the vesicle release rate in response to 4 *μ*A stimulation with different pulse durations, when the carrier frequency was fixed at 2 Hz. Maximum steady-state response amplitude gradually increased with longer pulses and plateaued around ~7–10 ms, depending on stimulus amplitude (figure 6(B)). A similar trend was observed in measurements of the RGC firing rate in rat retina stimulated subretinally ex-vivo (Mathieson et al 2012) as well as with visually evoked potentials measured in rats *in-vivo* (Lorach et al 2015b). As described previously (Werginz and Rattay 2016), increase in the stimulus amplitude initially leads to increased BC response, but then above certain threshold, the response starts decreasing (figure 6(C)). Such an upper threshold is expected when the membrane potential exceeds the reversal potential of calcium, which is in the range of 20–100 mV, depending on [Ca]_i_ (Werginz and Rattay 2016). Such non-monotonic responses also vary with pulse duration, as shown in figure 6(C). At very strong stimuli (>15 *μ*A), the maximum release rate reached the same level for all amplitudes and pulse durations—approximately 10% of the maximum level.

In summary, the modeled response of the BC to pulsatile electrical stimulation was generally in good agreement with the previously published experimental results. The model suggests that kinetics of the calcium channels together with the vesicle release dynamics in ribbon synapses capture the BC response characteristics observed in experiments with degenerate rat retina.

### 3.5. BC response to alternating gratings

Retinal (and cortical) response to alternating gratings is a well-established test of resolution and contrast sensitivity in animals (Harnois et al 1984) and in human infants (Dobson and Teller 1978, Sokol 1978). The grating period (or contrast) is decreased until the RGC (or cortical) response to the grating reversal vanishes into the noise. We used this test with natural and prosthetic vision ex-vivo (Lorach et al 2015b) and *in-vivo* (Ho et al 2019) in rats and demonstrated that the spatial resolution of prosthetic vision can match the pixel pitch (Lorach et al 2015b, Ho et al 2019). In rats, the resolvable grating stripe width in these measurements is about 10 times smaller than the average size of receptive field (20 *μ*m vs. 200 *μ*m). If BC responses would just linearly reproduce the illumination intensity and sum-up into the RGC, there would be no response to alternation since the average intensity over the receptive field much larger than the stripe width does not change much. What enables the RGC response in this case is adaptation—decrease of the response to a constant stimulus over time. Such a transient response to a step in continuous illumination (mimicking normal vision, albeit without the low-pass filtering by photoreceptors) is shown in the bottom row of figure 4(C). Transiency of the BC response is due to faster initial release of the vesicles from the primed pool, compared to sustained release from the slower pool. To model this effect, we summed the output from two distinct BCs into an RGC, as has been proposed by Demb et al (Demb et al 2001).

**Figure 7.**
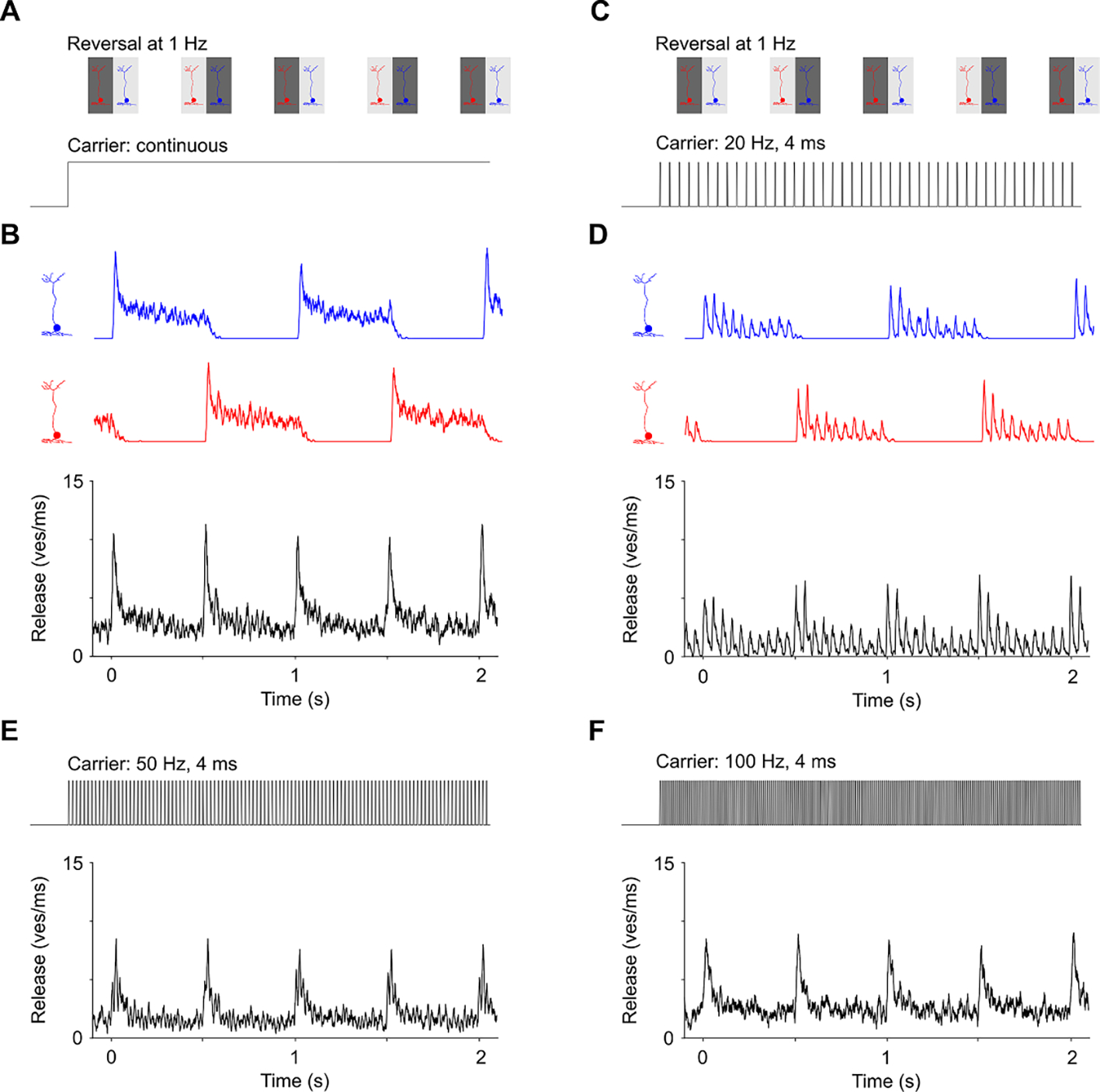
BC response to alternating grating stimulation. (**A**) Diagram illustrating stimulation with gratings alternating at 1 Hz (top row), when two different BCs correspond to the bright and dark stripes of the grating. (**B**) Responses from the two BCs (red and blue) are combined to simulate summation of the synaptic inputs in RGC (black). (**C**)-(**F**) Similar as (A)-(B) but for pulsatile electrical stimulation. The underlying carrier frequency was 20 (D), 50 (E) and 100 Hz (F) with 4 ms stimulus duration (bottom row in (C) and top row in (E)-(F)). All simulations were computed with an accelerated refill time constant of 0.5 s.

In the grating reversal paradigm, shown in figures 7(A)–(B), the total release rate is higher upon the grating reversal for each half-a-cycle, and it declines after that until the next reversal. Such compound signal explains the typically observed frequency doubling during stimulation with alternating gratings (Demb et al 2001, Lorach et al 2015a). The grating reversal is detectable until such oscillations decrease below the noise level, when stripes become smaller than the BC sub-units.

With electrical stimulation, we apply pulsed stimuli, and in order to prevent strong image flickering, which may interfere with the perception of the grating reversal, we operate at frequencies sufficiently high for flicker fusion. Patients with a subretinal implant could still see a little bit of flicker at 30 Hz, and no flicker at all at 60 Hz (Palanker et al 2020). In rats we typically operate at 40 Hz *in-vivo* (Ho et al 2019), while we limited the rep. rate to 20 Hz ex-vivo, due to the lower retinal temperature used to slow its metabolic demand. In this simulation, gratings were reversed at 1 Hz and a 20, 50 and 100 Hz carrier with 4 ms pulses was used (figures 7(c)–(F)). Each BC generated synaptic output when the pulsed electric field was applied to the corresponding BC, i.e. under the bright grating stripe but not under the dark stripe. Responses to the 20 Hz carrier showed transients at each grating reversal and each individual pulse led to a smaller transient release (figure 7(D)). With higher carrier frequencies, the response to the grating reversal became closer to that under continuous illumination (figure 7(B)). Like the responses with a continuous stimulus, responses with 50 and 100 Hz carrier are stronger right after the grating reversal, and decrease over time until the next reversal (figures 7(E)–(F)). At carrier frequency much below 20 Hz, there would be no significant attenuation of the pulse response, and hence such paradigm would not work for the grating acuity test.

Transient vesicle release in response to the grating reversal increased with the stimulus strength and then declined, when the stimulus amplitude exceeded the upper threshold, as shown in figure 6(D) for 50 Hz carrier frequency and grating reversal at 1 Hz. Both, the lower and upper stimulation thresholds decreased with increasing pulse duration, as shown in figure 6(D) for 1, 4, 10 ms and CW stimulation.

## 4. Discussion

Photoreceptors operate within about 8–10 orders of magnitude in luminance, with this range divided between rods specializing in scotopic, and cones—in photopic vision. Their response to increasing brightness follows a sigmoidal curve, with about 2 orders of magnitude in the center of the range providing a nearly linear rise, and saturating towards zero on the lower end and towards maximum on the higher end. Photoreceptors adjust their amplification mechanisms (together with the pupillary response) such that their response curve centers around the average brightness level. In retinal prosthetics, this function is performed by the camera which adapts to the ambient illumination, so that the brightness of the images projected onto the photovoltaic pixels matches their dynamic range for retinal stimulation.

As shown in figure 6, dynamic range of the BC response to electrical stimulation (from the stimulation threshold to saturation) is much narrower than the natural 2 orders of magnitude (Pelli and Bex 2013). This is likely to result in reduced temporal contrast sensitivity of prosthetic vision, as was observed ex-vivo and *in-vivo* (Ho et al 2018).

Horizontal cells (HCs), wired to the terminals of photoreceptors, inhibit the photoreceptors’ output into BCs based on the average luminance level within their receptive fields (antagonistic center-surround) and thereby enhance the spatial contrast. Upon demise of photoreceptors in retinal degeneration, HCs become disconnected from the retinal circuit, so their function should also be performed externally—by contrast enhancement between the camera and the stimulating array.

To preserve the remaining retinal signal processing as much as possible when the lost photoreceptors are replaced with photovoltaic pixels, they should be coupled into the secondary retinal neurons in a manner that is (1) spatially selective, and (2) retain temporal dynamics similar to natural vision.

### 4.1. Spatial selectivity

Spatial selectivity of electrical stimulation can be enhanced either by providing local return electrodes around each pixel to confine the electric field, or by sequential activation of the pixels so that their electric fields do not add up and hence the electrical crosstalk is reduced. Temporal multiplexing is limited by the ratio of the frame duration to pulse duration. For example, with a frame rate of 33 Hz and pulse duration of 10 ms, only 3 groups of electrodes can be activated sequentially. Since penetration depth of the electric field generated by a flat bipolar pixel is similar to its radius, pixels smaller than 40 *μ*m cannot effectively stimulate the whole INL (Ho et al 2019). Pillar electrodes can center the electric field in the middle of the INL (figure 3), but this decreases the selectivity between stimulation of BCs and ACs, located in the top strata of INL and (some) displaced into the ganglion cell layer. Honeycomb implants provide optimal balance between the lateral confinement of the electric field and axial selectivity between the top and bottom of the INL (figure 3). With honeycombs, the penetration depth of the electric field is decoupled from pixel width, meaning that this geometry is scalable down to cellular sizes.

### 4.2. Temporal filtering and rectification in BCs

Naturally, photoreceptors provide a continuous influx of glutamate into BCs, modulated by light intensity. In electrical stimulation, the applied stimuli should be pulsed and biphasic due to the requirement of charge balance. To make the effect of pulsed stimulation on the retinal network as close to that of a continuous stimulus as possible, we operate at frequencies above the flicker fusion. Since the resting membrane potential is negative, cells respond to an alternating current as rectifiers—they only allow cations inflow when ion channels open during depolarization, and the ion concentration is restored after the stimulus by ion pumps and leakage currents. Patch clamp experiments demonstrated pulsatile depolarizing response to extracellular electrical stimulation in retinal BCs (Walston et al 2018) accompanied by the glutamate release from their axonal terminals (Margalit and Thoreson 2006).

Due to the relatively slow pump-out of Ca from BCs, its intracellular concentration under high frequency stimulation increases, and the amplitude of inter-pulse oscillations decreases, which represents one aspect of the low-pass temporal filtering, as shown in figure 4(B). Decreasing amplitude of the BC response with increasing frequency of pulsatile extracellular electrical stimulation has been observed in direct patch clamp recordings (Walston et al 2018). The second phase of temporal filtering in BCs occurs due to specific vesicle release dynamics in the ribbon synapses. When the readily available pool of vesicles is exhausted after the initial rapid response, the sustained rate becomes much lower and its dynamics is much slower (the transient response is ~50x larger than the sustained response, figure 4(C) and (Singer and Diamond 2006)). Therefore, with increasing stimulation frequency, the release rate dynamics becomes more and more similar to that observed with continuous illumination, as shown in figure 4(C). Such temporal filtering represents not only flicker fusion, but also adaptation to a static input (response to a constant stimulus diminishes over time (Suh and Baccus 2014)). Photoreceptors also represent low-pass temporal filtering, and therefore flicker fusion in healthy retina occurs faster than in degenerate retina, as shown in figure 5(D). For this reason, we operate typically at 40 Hz in rodents *in-vivo*, and human patients with photovoltaic implants reported that they see much less flickering at 30 Hz than at 10 Hz, and no flickering at all at 60 Hz (Palanker et al 2020).

Another important aspect of the ribbon synapse is the rectifying nonlinearity, which arises from the fact that the vesicle release cannot be negative. Thus, the vesicle release rate initially shoots up upon a rapid increase in membrane potential and then drops to a much lower sustained rate, but does not turn negative during the falling edge of the voltage pulse. Such temporal filtering and rectification enable the nonlinear summation of inputs from multiple bipolar cells (sub-units) into an RGC, which explains the response to alternating gratings with the stripe width much smaller than the average size of its receptive field, as shown in figure 7, and measured experimentally in (Lorach et al 2015a).

The physiological properties of the ribbon synapses contribute to multiple aspects of retinal neural computations (reviewed in (Lagnado and Schmitz 2015)), and preservation of their response dynamics under high frequency electrical stimulation should retain these aspects of the natural signal processing. In addition to flicker fusion, adaptation to static images and non-linear summation of sub-units (Lorach et al 2015a), we see preservation of several other important aspects of the retinal signal processing, likely due to properly functioning amacrine cells. One preserved feature is the antagonistic center-surround organization of the RGC receptive fields in prosthetic vision (Ho et al 2018) and the other—inhibitory responses of OFF RGCs (figure 3 in (Ho et al 2019)).

### 4.3. Limitations of selectivity and their potential consequences

One potential limitation of the ‘electronic photoreceptors’ is the indiscriminate activation of ON- and OFF-cone BCs. In ex-vivo recordings, we see much fewer OFF-responding RGCs than ON, unlike in natural vision in rats, where the split is close to equal (Ho et al 2018). In addition, patients with our implants (PRIMA, Pixium Vision) report perceptions faithfully representing the polarity of the visual stimuli, such as bright letters on dark background (Palanker et al 2020). One reason why indiscriminate stimulation of the ON and OFF BCs elicits correct visual percepts may be the role of rod BCs. In natural day vision, rod BCs are mostly saturated and do not play a major role in modulating the RGC firing rate. Electronic photoreceptors however, likely stimulate rod and cone BCs to the same extent. Since there are only ON-rod BCs, which are coupled into the cone pathway by enhancing the ON and inhibiting the OFF signals, they correctly represent the contrast of the excitatory input into BCs. Another reason could be the much higher spontaneous firing rate of the OFF RGCs than ON in degenerate retina (Sekirnjak et al 2011). With the same number of elicited spikes, the signal-to-noise ratio in the quiet ON pathway will be much higher than that in the noisy OFF RGCs, and hence the ON responses may perceptually dominate.

Another important factor for selective stimulation of single BC subtypes might be the distribution of different subclasses of calcium channels in BC terminals. Here, we modeled a calcium L-type channel as it has been shown to be driving vesicle release from BC terminals (reviewed in (Wan and Heidelberger 2011)). Another class—the T-type calcium channels—has also been found in BCs and are involved in neurotransmitter release (Pan et al 2001). In contrast to L-type, the T-type channels are characterized by lower activation thresholds and slower dynamics. Therefore, retinal implants could use lower current to activate BCs expressing T-type channels, which would allow selective stimulation of these BC types over others. Also, the larger activation time constants of T-type channels would slow down BC responses, thereby increasing chronaxie and improving selectivity of BCs activation over RGCs even further. Therefore, BC chronaxies presented in figure 2(A) should be considered as a lower bound. Since little is known about the distribution of different types of calcium channels in BCs, this issue requires further exploration.

Lastly, the whole concept of electronic replacement of the lost photoreceptors in atrophic AMD relies on the assumption of preservation of the inner retinal cells and circuits to a large extent. Even though the first clinical results with this approach are very encouraging (Palanker et al 2020), the extent of the retinal preservation, as well as the effect of retinal migration into the honeycomb-shaped implants remains to be verified experimentally.

### 4.4. Model limitations

In this study we assumed the retinal resistivity to be 1000 Ω cm, which is in the mid-range of the previously reported values (Karwoski et al 1985, Greenberg et al 1999, Kasi et al 2011). The good agreement between the computed and experimentally measured RGC thresholds (figure 2(A)) adds support to the assumed retinal resistivity.

Simulated BC S-D curves had lower chronaxies than observed in experiments (~3.5 vs. 7 ms in (Boinagrov et al 2014)). Thresholds matched well for short pulse durations, however, for pulses longer than 2 ms, the simulated and experimental data diverged (figure 2(A)). By adjusting the model parameters, such as extracellular resistivity, calcium channel density as well as the threshold criterion, the rheobase can be matched. However, such modifications would only shift the S-D curve along the vertical axis, but will not change the chronaxie values. The mismatch of chronaxie might be related to post-synaptic processing on RGCs, which is not included in the present model. Post-synaptic glutamate receptors, as well as the dendritic ion channels, shape the BC input and affect the RGC spiking, and therefore can extend the corresponding chronaxies. Furthermore, since RGCs are also depolarized to some extent during BC stimulation, such sub-threshold activation might lower the BC-mediated thresholds.

Multiple mechanisms are involved in pumping calcium ions out of the intracellular space, and many aspects of these processes are still not very well understood. Here, we modeled extrusion of calcium from the intracellular space with a single time constant of 50 ms. This value is smaller than the hundreds of milliseconds time constants observed with calcium indicators (Burrone et al 2002). Since the latter have fast onset but a slow decaying phase of the response signal (Stringer and Pachitariu 2019), these observations result in overestimation of the Ca extrusion time constant. Direct whole-cell recordings in neurons yielded [Ca]_i_ decay times in the range of tens of milliseconds (Dittman and Regehr 1998).

Recently, it has been reported that BCs can initiate calcium-driven spikes in response to electrical stimulation of the retina (Walston et al 2018). Since the origin as well as kinetics of these calcium spikes are still not fully understood, we did not model them in the present study. The reported transient depolarizations had an amplitude of only 10–20 mV, which might not be enough to trigger substantial synaptic release. However, measurements in (Walston et al 2018) were performed at the soma, whereas calcium spikes are thought to be initiated within the axon (terminals) of BCs. It will be interesting to learn in the future the potential effect of calcium spikes on the performance of subretinal prostheses.

## 5. Conclusions

The modeled BC-mediated retinal responses were in general in good agreement with previously published experimental results. Kinetics of the calcium pumps and of the neurotransmitter release from ribbon synapses, which underpin the BC’s temporal filtering and rectifying functions, allow mimicking the natural BC response to continuous visual stimuli using high frequency pulsatile electrical stimulation. Such replacement preserves many features of the natural retinal signal processing such as flicker fusion, adaptation to static stimuli and non-linear summation of subunits in the RGC receptive field. Selectivity of the BC stimulation while avoiding direct activation of the downstream neurons is further improved using local return electrodes. Selective stimulation of BCs using proper spatial and temporal modulation of the electric field may allow highly functional restoration of sight.

## Supplementary Material

Supplementary Material

## Figures and Tables

**Table 1.** Ion channel densities on the membrane of the model RGC. Values are based on Fohlmeister et al (Fohlmeister et al 2010) with minor modifications. All values are given in mS/cm^2.^

	Dendrites	Soma	Soma-AIS	AIS	Axon

gNa	50	70	175	350	100
gK	35	35	90	175	50
gCa	1	0.75	0.75	0.75	0.75
gK,Ca	0.11	0.05	0.05	0.05	0.25
gL	0.5	0.5	0.5	0.5	0.5

**Table 2.** Ion channel densities on the membrane of the model BC. All values are given in mS/cm^2.^

	Dendrites	Soma	Axon	Terminals

gCa	0	0	0	0.5
gL	0.5	0.5	0.5	0.5
