# Supplementary material for: On optimal coupling of the ‘electronic photoreceptors’ into the degenerate retina
Source: J Neural Eng. Author manuscript; Available in PMC 2024 Mar 18. (PMC10948023; doi:10.1088/1741-2552/aba0d2)
Supplement: Supplementary Material [file NIHMS1971807-supplement-Supplementary_Material.pdf]

Supplementary Material

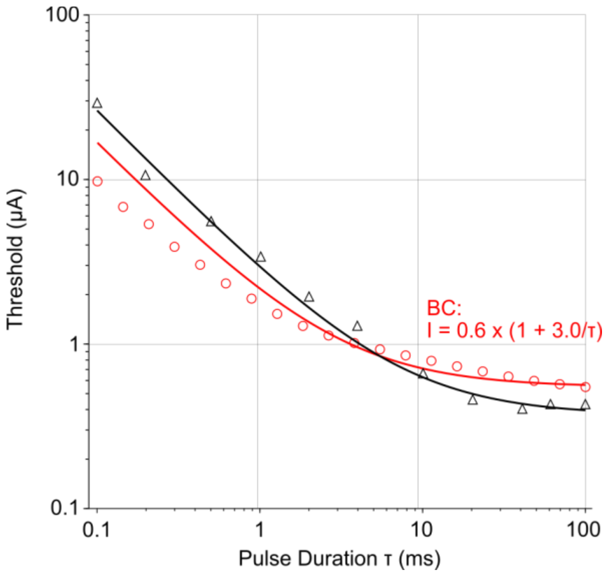

Figure S1: Strength-Duration (S-D) curve for BC activation based on the threshold defined as the $[Ca]_i$ level exceeding 3 μM. Calculated vales are shown by circles, experimental data from (Boinagrov et al., 2014) - by triangular markers, and solid lines show Weiss equation fit into each dataset.

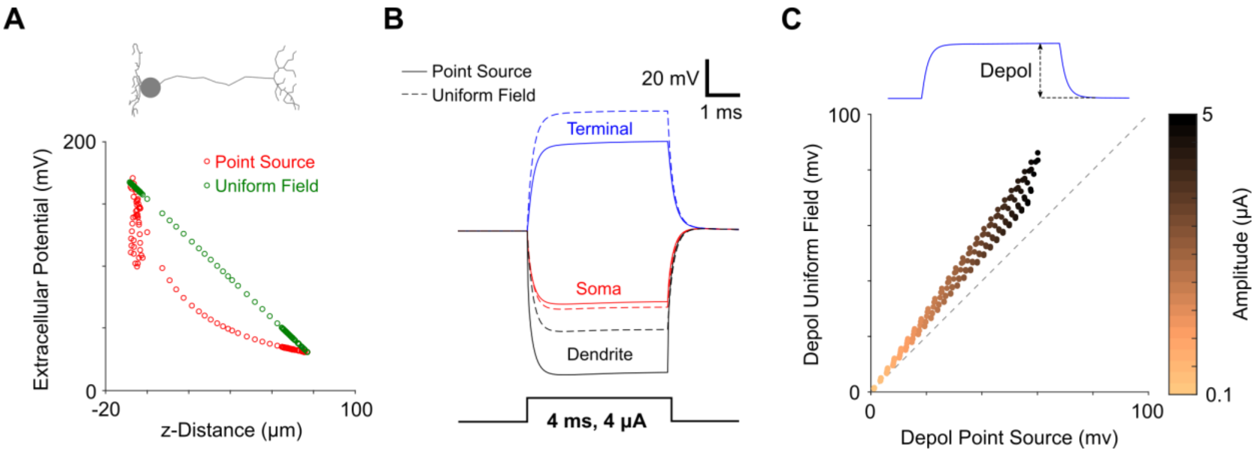

Figure S2: S**timulation by point source and uniform field.** (**A**) Extracellular potential vs. the z-distance of each compartment of the BC (morphology shown on top) for point source (red) and uniform field (green) stimulation. (**B**) Typical traces of the membrane potential over time for the somatic (red), one terminal (blue) and one dendritic compartment (black) for stimulation with a point source (solid lines) and uniform field (dashed lines). The pulse (4 ms, 4 μA) is indicated at the bottom. (**C**) Maximum membrane depolarization in synaptic terminals (diagram on top) for stimulation amplitudes ranging from 0.1 to 5 μA for point source vs. uniform filed stimulation.
